# Effects of low molecular weight peptides from monkfish (*Lophius litulon*) roe on immune response in immunosuppressed mice

**DOI:** 10.3389/fnut.2022.929105

**Published:** 2022-09-21

**Authors:** Zhexin Ren, Fei Yang, Sijia Yao, Lijun Bi, Guanqin Jiang, Ju Huang, Yunping Tang

**Affiliations:** ^1^Zhejiang Provincial Engineering Technology Research Center of Marine Biomedical Products, School of Food and Pharmacy, Zhejiang Ocean University, Zhoushan, China; ^2^Hangzhou Women's Hospital (Hangzhou Maternity and Child Health Care Hospital), Neonatal Intensive Care Unit, Hangzhou, China; ^3^College of Food Science and Biotechnology, Zhejiang Gongshang University, Hangzhou, China; ^4^Key Laboratory of Health Risk Factors for Seafood of Zhejiang Province, Zhejiang Ocean University, Zhoushan, China

**Keywords:** *(Lophius litulon)* roe, low molecular weight peptides, immunosuppression, immunomodulatory, NF-κB and MAPK pathways

## Abstract

This study aimed to investigate the immunomodulatory activation of low-molecular-weight peptides from monkfish (*Lophius litulon*) roe (named MRP) on cyclophosphamide (CTX)-induced immunosuppressed mice. Our results indicated that MRP (100 mg/kg/d BW) could significantly increase the body weight and immune organ index, and improve the morphological changes in the spleen and thymus of mice. These effects subsequently enhance the serum levels of interleukin (IL)-6, IL-1β, tumor necrosis factor (TNF)-α, and immunoglobulin (Ig) A, IgM, and IgG. Furthermore, MRP could also improve CTX-induced oxidative stress, and activate the NF-κB and MAPK pathways in the spleen tissues. The findings reported herein indicate that MRP has a good immunomodulatory activation toward immunosuppressed mice, hence can potentially be developed as an immune adjuvant or functional food.

## Introduction

The immune capacity of the human body is affected and challenged by many factors due to changing lifestyles (1, 2). Diseases, aging, obesity, improper diet, mental stress, and unhealthy living habits can all lead to a decline in immunity and result in various conditions (3, 4). The immune system regulates multiple physiological processes in the human body and plays an important role in keeping human health (5, 6). Traditional immunomodulators, such as glucocorticoid, cyclosporine, tacrolimus, and levamisole (7–9), regulate the immune response. However, these drugs are expensive, have toxic side effects on the body, and are often unsuitable for chronic or preventive use. Therefore, searching for novel and non-cytotoxicity immunomodulators with high activity and few side effects is highly important.

Bioactive peptides containing 2–20 amino acids can exert various physiological effects, such as immunoregulation, antioxidant, antibacterial, antitumor, and hepato-protective impact (10–12). These peptides have been gaining increasing attention recently due to their bioactivity, high safety and bioavailability, and diverse physiological functions. Many immunomodulatory peptides have also been synthesized from plant- and animal-derived proteins by enzymolysis. For example, Wen et al. (13) isolated several peptides from soybean protein and identified 46 immunomodulatory peptides, while Yu et al. (14) purified an immunomodulatory peptide (Lys-Ser-Pro-Leu-Tyr) from *Hericium erinaceus*. In our previous studies, we purified an immunomodulatory peptide from *Cyclina sinensis* and found that it has excellent immunomodulatory activities both *in vitro* and *in vivo* (15, 16). These findings suggest that immunomodulatory peptides derived from plants and animals can potentially be used as nutritional supplements to improve immune function in humans.

Monkfish (*Lophius litulon*) is mainly found in the Western North Pacific and coastal waters of China (17). There are several reports on the preparation of active peptides from monkfish. For example, Hu et al. (11) prepared antioxidant peptides from the protein hydrolysate of monkfish muscle, while Tian et al. (17) optimized the extraction factors of antioxidant peptides from monkfish muscle, and investigated their role in H_2_O_2_-induced lesion. Ye et al. (12) investigated the amelioration effect of monkfish peptides toward high fat diet-induced hepatic steatosis in mice. However, there is no report on the preparation of immunomodulatory peptides from monkfish. Fish roe is a by-product of fish processing, and is rich in proteins, unsaturated fatty acids, phospholipids, essential amino acids and minerals (18, 19). However, monkfish roe cannot be effectively processed and utilized because it does not meet the processing requirements of caviar. Thus, the roe is typically discarded, resulting in resource waste and subsequent environmental pollution. For the efficient utilization of this resource, low-molecular-weight (LMW) peptides from monkfish roe (named MRP) were prepared through enzymolysis, ultrafiltration, and lyophilization. This study aimed to determine the immunomodulatory effect of MRP using immunosuppressed mice and elucidate the underlying immunomodulation mechanism. Our findings serve as a reference for the efficient utilization of fish roe, and suggest the potential of MRP application as an adjuvant to functional food in enhancing immunity.

## Materials and methods

### Materials and reagents

Monkfish roe was purchased from Zhoushan International Aquatic Center (Zhejiang, China). Cyclophosphamide (CTX) was purchased from Aladdin (Shanghai, China). The hematoxylin-eosin (H&E) staining kit was purchased from Beyotime (Shanghai, China). Tumor necrosis factor (TNF)-α, interleukin (IL)-6, and IL-1β ELISA kits were purchased from Boster (Wuhan, China). Immunoglobulin (Ig)A, IgM, and IgG ELISA kits were purchased from Yilai Ruite Biotechnology (Wuhan, China). Commercial kits for malondialdehyde (MDA), total antioxidant capacity (T-AOC), glutathione peroxidase (GSH-Px), superoxide dismutase (SOD), and catalase (CAT) were purchased from Jiancheng (Nanjing, China). Antibodies were purchased from Beyotime (Shanghai, China) or Cell Signaling Technology (Boston, MA, USA).

### Preparation of bioactive peptides from monkfish roe

Bioactive peptides from monkfish roe were prepared using stepwise enzymatic hydrolysis described previously (20, 21). The roe was defatted using 95% ethanol and homogenized. The reaction system (roe: water = 1:10, w/w) was adjusted to pH 2.0 and then hydrolyzed using 1,500 U/g pepsin for 4 h at 37°C. The reaction system was then adjusted to pH 8.0 and hydrolyzed using 1,500 U/g trypsin for 4 h at 37°C. The reaction system was subsequently heated, cooled and centrifuged at 8,000 g for 10 min. Monkfish roe peptides (MRP) with a molecular weight (MW) < 1 kDa were obtained from the supernatant by ultrafiltration using a 1 kDa membrane and then freeze-dried for further studies.

### Determination of molecular weight and amino acid composition

The MW of MRP was analyzed as described in our previous studies (20, 21), using Agilent 1260 Infinity HPLC with a TSK gel G2000 SW_XL_ column (7.8 × 300 mm, 5 μm) at 220 nm. The MW standards used were cytochrome C (12,355 Da), aprotinin (6,511 Da), bacitracin (1,422 Da), and tetrapeptide GGYR (451 Da). A standard curve of the linear relationship between the retention time and average log MW (lg MW) was used to calculate the MW distribution of MRP. Amino acid analysis was performed according to Tang et al. (22) using a Hitachi L-8900 amino acid analyzer (Tokyo, Japan).

### Animals and design

**Figure 1:**
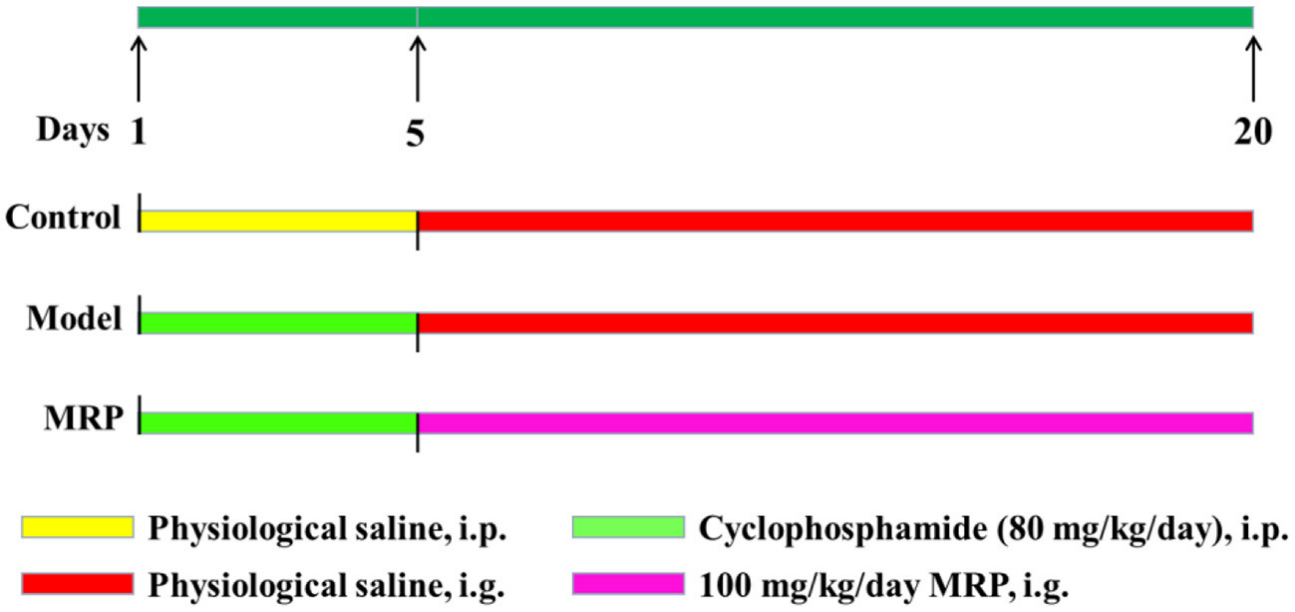
The experimental protocol was performed in the present study.

Male ICR mice (20 ± 2 g, 6–8 weeks) were purchased from the Zhejiang Academy of Medical Sciences. After 7 days of adaptive feeding, the mice were randomly divided into three groups (*n* = 10 per group): the control (saline solution), model (80 mg/kg/d BW CTX), and MRP (100 mg/kg/d BW MRP). The control mice received gavages with a saline solution daily for 20 days, and the remaining two groups were firstly injected with CTX (80 mg/kg/d BW) daily for 5 days and then saline solution or MRP (dissolved in saline solution) daily for 15 days (Figure 1). After 24 h of the last drug administration, the mice were anesthetized with chloral hydrate (10%) and sacrificed by cervical dislocation.

### Body weight and immune organ index determination

The body weight of mice was recorded daily, and the spleen and thymus indices were calculated as follows:


$$\begin{array}{l} \text{spleen or thymus index }\left(\text{mg}/\text{g}\right)=\text{spleen or thymus weight }\left(\text{mg}\right)\\ /\text{ body weight }\left(\text{g}\right) \end{array}$$

### Histopathological evaluation

The spleen and thymus cross-sections were fixed using 4% paraformaldehyde 4% and embedded in 10% paraffin 10%. The H&E staining was performed as described previously (15, 23), and micrographs were taken using a CX31 light microscope (Olympus, Japan).

### Biochemical determination

The blood was collected from eyeball extirpation and centrifuged at 6,000 × g for 3 min at 4°C to obtain serum. The levels of TNF-α, IL-6, and IL-1β in serum were determined using Boster's ELISA kits. The levels of IgA, IgM, and IgG in serum were determined using Yilai Ruite's ELISA kits.

### Oxidative stress index determination

The spleen tissues were ground in saline solution, and then centrifuged at 12,000 × g for 3 min at 4°C. The levels of MDA, T-AOC, CAT, SOD, and GSH-Px in spleen tissues were measured using Jiancheng's commercial kits.

### Western blot analysis

The spleen tissues were ground, and the supernatant was collected by centrifugation. The protein concentration was determined using a BCA assay kit (Solarbio, Beijing, China). The protein (30 μg) was then separated using a 12% SDS-PAGE gel, and a western blot was performed as described in our previous studies (20, 24). The primary antibodies used were as follows: GAPDH (AF1186, 1:1000), TLR 4 (AF8187, 1:1000), NF-κB p65 (AF0639, 1:1000), IKKα (AF0198, 1:1000), IKKβ (AF7200, 1:1000), IκBα (AF5204, 1:1000), and p-IκB (AF1870 1:1000) were purchased from Beyotime. JNK (#9252, 1:1000), p-JNK (#4668, 1:1000), ERK (#4695, 1:1000), p-ERK (#4370, 1:1000), P38 (#8690, 1:1000) and p-P38 (#4511, 1:1000) were purchased from Cell Signaling Technology.

### Statistical analysis

The results were expressed as the mean ± standard deviation (SD). Data analysis was performed using SPSS 24.0 (SPSS Inc., Chicago, IL, USA). One-way analysis of variance (ANOVA) and LSD *post hoc* test were used to analyze the differences among groups, and *P* < 0.05 was considered statistically significant.

## Results and discussion

### MW distribution of MRP

**Figure 2:**
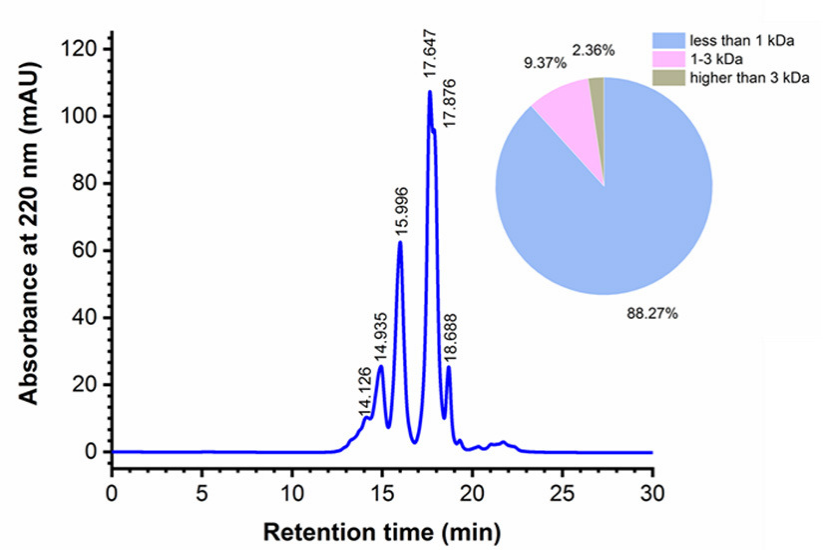
Molecular weight (MW) distribution of MRP.

The yield of MRP was 33.55 ± 1.58% on the basis of monkfish roe dry weight, and the MW profile of MRP was determined using gel permeation chromatography (Figure 2). The calibration equation was obtained as the retention time (Rt) against the logarithm of the standards MW (LgMW): LgMW = −0.2183Rt + 6.4137, *R*^2^ = 0.9994. Most peptides had MWs of < 1 kDa and accounted for 88.27% of the total MRP. Peptides with MW in the range of 1–3 kDa accounted for 9.37% of the total MRP, whereas those with MWs higher than 3 kDa only accounted for 2.36% of the total MRP. The results indicate the effectiveness of the stepwise enzymatic hydrolysis in isolating LMW peptides.

LMW peptides exhibit stronger immunomodulatory effects (23, 25). Moreover, ingesting LMW peptides leads to enhanced intestinal absorption, resulting in higher bioavailability (26, 27). Yu et al. (23) showed that LMW peptides (< 3 kDa) from *Nibea japonica* skin induced the highest proliferation of RAW264.7 cells compared with other fractions (3–5 kDa, 5–10 kDa, and > 10 kDa). Xu et al. (25) showed that LMW peptides (< 1 kDa) from *Stolephorus chinensis* induced the highest proliferation of RAW264.7 cells, and subsequently isolated an immunoregulatory peptide, Tyr-Val-Met-Arg-Phe, from the LMW peptides. In this study, 88.27% of the total MRP had smaller peptides having MWs < 1 kDa, indicating their promising bioactivities. Therefore, the immunomodulatory activities of these peptides were determined in subsequent experiments.

### Amino acid composition

**Figure 3:**
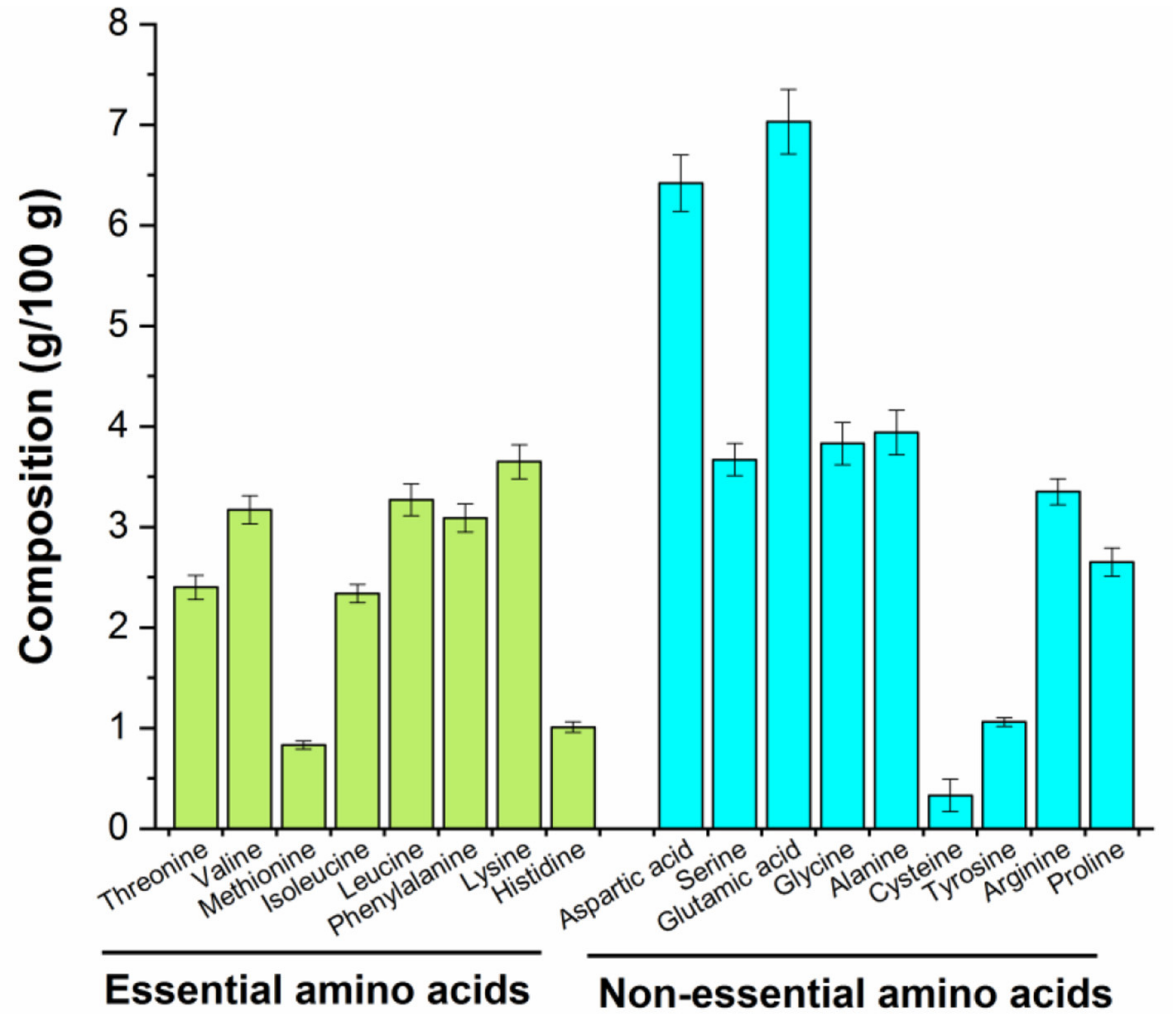
Amino acid composition of MRP.

The biological activity of peptides is mainly related to their amino acid composition (28). The amino acid composition of MRP is listed in Figure 3. Previous studies suggest that Arg, Glu, or phosphoserine residues may be recognized by receptors on the surface of immune cells that regulate the peripheral immune system (28, 29). The contents of Arg, Glu, and Ser in MRP were found to be 6.44, 13.51, and 7.05%, respectively. Moreover, positively charged amino acids (Lys, Arg, and His) may also interact with immune cells (29), and the content of these positively charged amino acids in MRP was determined to be 15.39%. Therefore, MRP may exert immunomodulatory effects because of the high content of Glu, Ser, and positively charged amino acids.

### Effect of MRP on body weight and immune organ index

**Figure 4:**
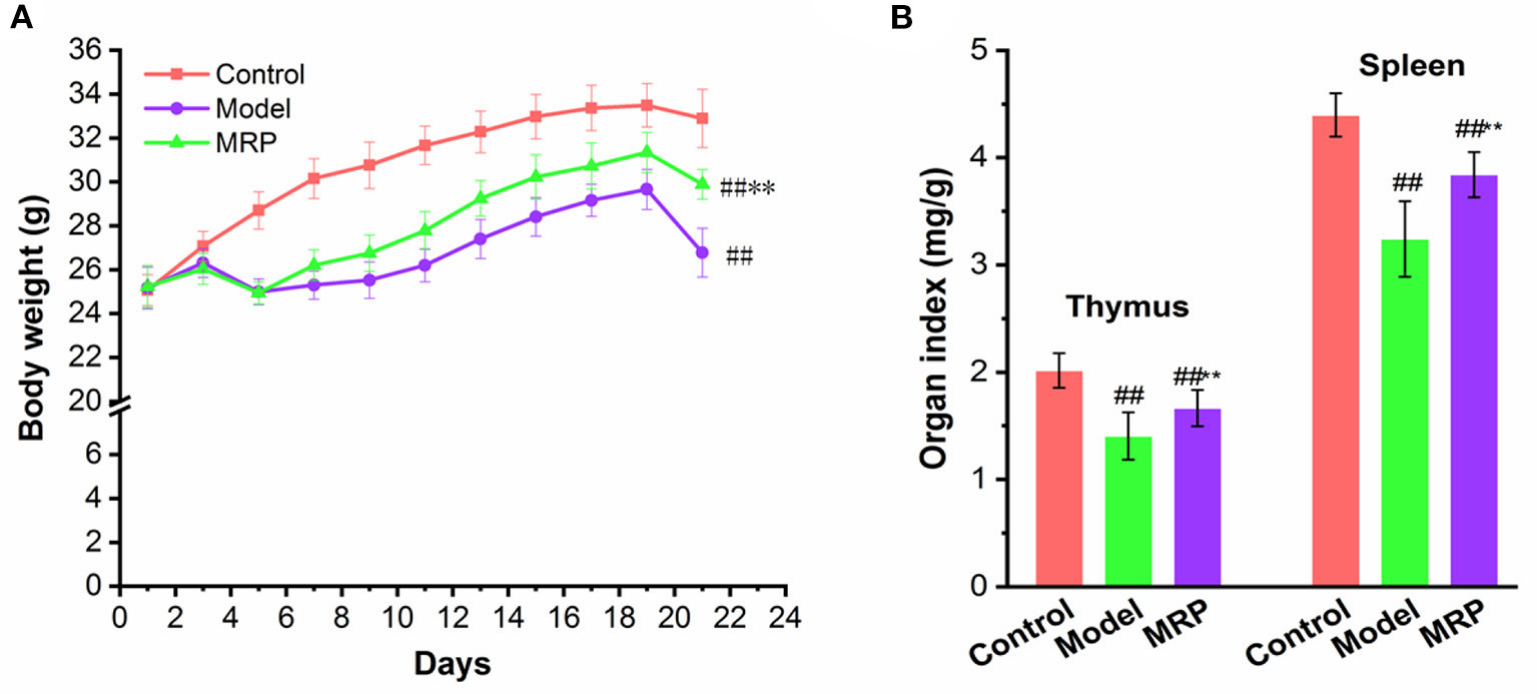
Effect of MRP on body weight **(A)** and immune organ index **(B)** in CTX-treated mice (*n* = 10). ^##^*P* < 0.01 vs. Control; **P < 0.01 vs. Model.

Our results showed that CTX significantly reduced body weight as compared to the control (Figure 4, *P* < 0.01). This finding is consistent with previous reports that the body weight can reflect the immunosuppression mice's growth status (15, 30). MRP treatment significantly increased the final average body weight of mice relative to the model (Figure 4A, *P* < 0.01), indicating that MRP could attenuate CTX-induced body weight loss. In addition, the thymus and spleen are important immune organs, and the immunomodulatory activities are closely related to changes in immune organ index (31, 32). As shown in Figure 4B, CTX treatment considerably reduced the thymus and spleen index of mice, as compared to the control (*P* < 0.05 or *P* < 0.01), indicating that the immunosuppressed mice model was successfully established. Meanwhile, MRP treatment significantly increased the thymus and spleen index (*P* < 0.05), suggesting that MRP treatment attenuated CTX-induced immune organ atrophy. Similar to previous studies (23, 33), LMW peptides from *Nibea japonica* skin and muscles could enhance the body weight and organ index in immunosuppression mice.

### Histopathological evaluation

**Figure 5:**
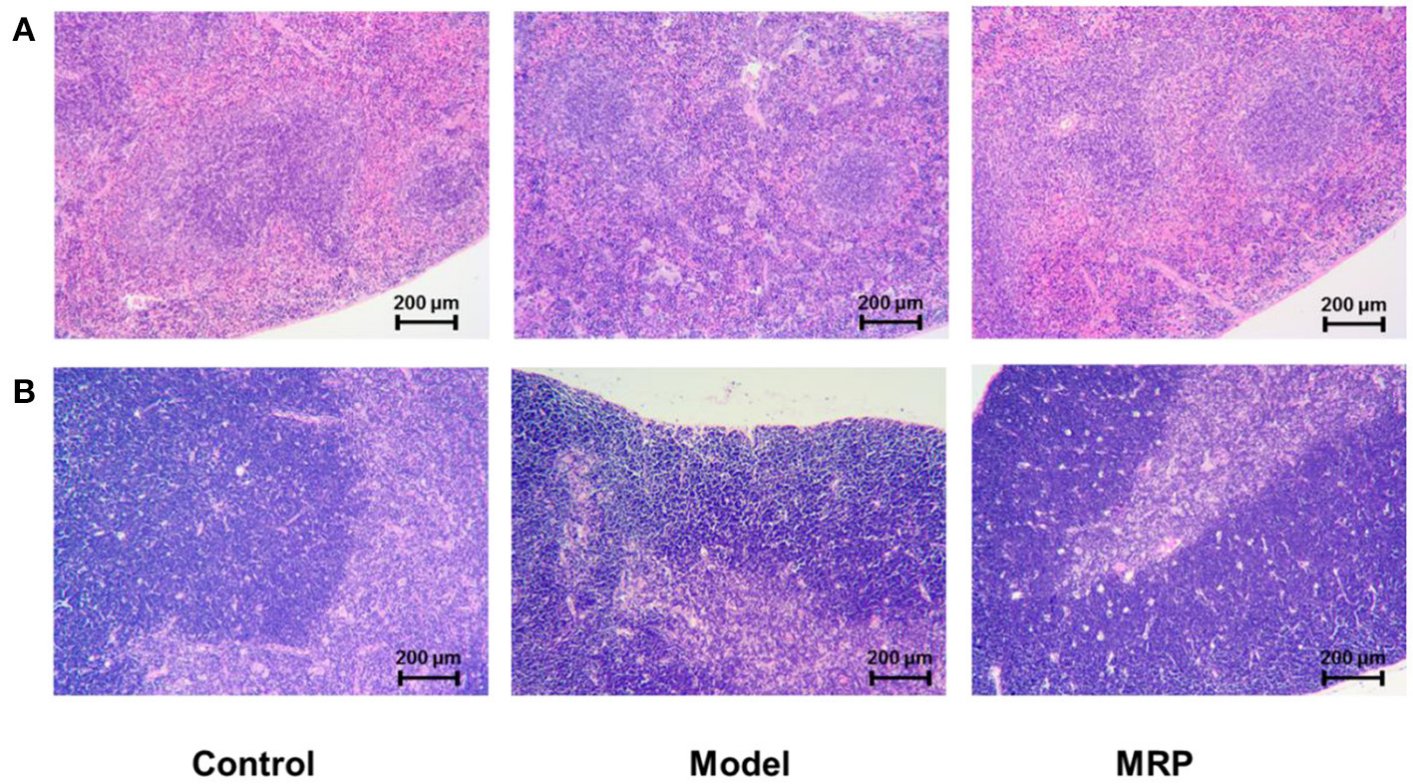
Histomorphology of the spleen **(A)** and thymus **(B)** in mice (×100).

H&E staining was used to observe morphological changes in immune organs (34) in mice's spleen and thymus (Figure 5). When treated with CTX, the boundary between the white and red-pulp of the spleen in the model was unclear, but the mature lymphocytes decreased with a certain amount of inflammatory cells. After treatment with MRP, the spleen's white and red-pulp boundaries became visible, and the number of inflammatory cells decreased compared to the model (Figure 5A). In addition, when treated with CTX, the thymus cortex was atrophied, the medulla was increased, and the thymus corpuscle decreased compared to the control (Figure 5B). After treatment with MRP, the thymus cutaneous pulp boundary became visible with a thicker cortex, and increased thymus corpuscle. Overall, the histopathological observation indicates that MRP could attenuate spleen and thymus injury in immunosuppression mice.

### Serum cytokines and immunoglobulins levels

**Figure 6:**
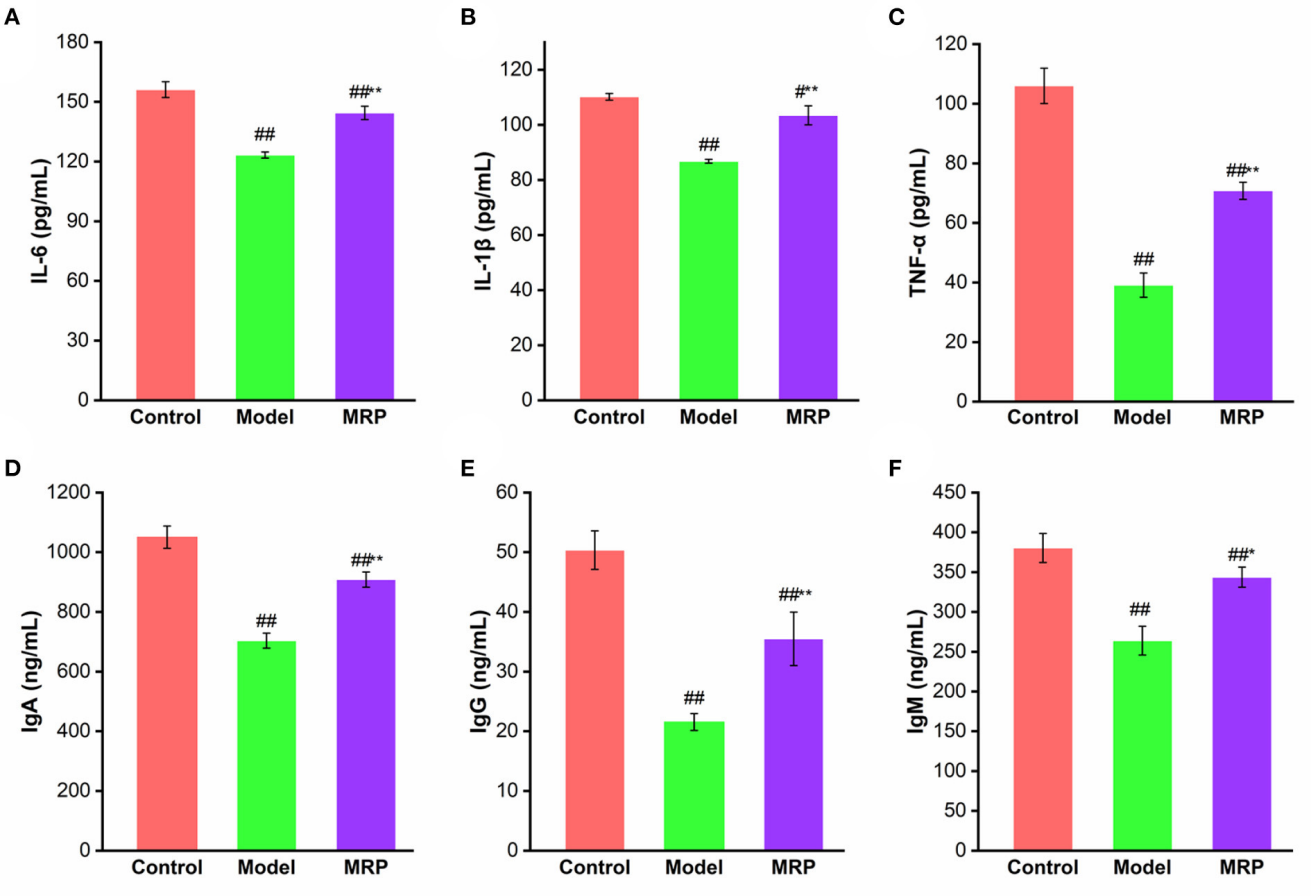
Effect of MRP on serum levels of IL-6 **(A)**, IL-1β **(B)**, TNF-α **(C)**, IgA **(D)**, IgM **(E)**, and IgG **(F)** (*n* = 10). ^#^*P* < 0.05 and ^##^*P* < 0.01 vs. Control; *P < 0.05 and **P < 0.01 vs. Model.

Because cytokines and immunoglobulins are important parts of the immune response and play vital roles in the organism's immune (30, 35). IL-6, IL-1β, and TNF-α are common markers that indicate the immunoregulatory activities of peptides (33). IgA is an antibody that plays an important role in mucosal immunity, and IgG is the most abundant antibody subtype in the body, while IgM could activate the complement system and strengthen phagocytosis in the presence of complement and macrophages (23). Therefore, the effects of MRP on the secretion of IL-6, IL-1β, TNF-α, IgA, IgM, and IgG in serum were evaluated. The serum levels of IL-6, IL-1β, TNF-α, IgA, IgM, and IgG significantly decreased after treatment with CTX (Figure 6; *P* < 0.01), confirming findings of previous research (34, 36). When treated with MRP, the levels of the cytokines and immunoglobulins were significantly enhanced compared with the model (*P* < 0.05 or 0.01), indicating that MRP could attenuate immunosuppression in mice by increasing the production of these proteins. Other studies have also shown immune enhancement by increasing the secretion of cytokines and immunoglobulins against CTX-induced mice immunosuppression (16, 37), corroborating our current findings.

### Antioxidant activity of MRP in spleen

The dynamic balance between the oxidative and antioxidant states of the body plays a critical role in safeguarding an organism's health (38). MDA reflects the degree of lipid peroxidation after tissue damage and oxidative stress (24), while T-AOC reflects the capacity of enzymatic and non-enzymatic antioxidant defenses (20). In addition, GSH-Px, SOD, and CAT play important roles in cellular defense against the adverse effects caused by free oxygen radicals (20, 24). CTX can increase the MDA contents, and decrease the T-AOC levels and antioxidant enzymes activities, thus destroying the host's redox balance and normal metabolism (39). As shown in Table 1, MDA content in the spleen was remarkably increased (*P* < 0.01), while the T-AOC levels (*P* < 0.01) and antioxidant enzyme activities in the spleen were significantly decreased in the model (*P* < 0.01). After treatment with MRP, the MDA contents significantly reduced (*P* < 0.05), while the T-AOC levels (*P* < 0.05), CAT (*P* < 0.01), SOD (*P* < 0.05) and GSH-Px (*P* < 0.05) activities significantly increased compared to the model. These results suggest that MRP could improve CTX-induced oxidative stress in the spleen tissues and maintain the host's redox balance for normal metabolism.

**Table 1:** Effect of MRP on the oxidative stress indices of the spleen.

**Group**	**Control**	**Model**	**MRP**
MDA (nmol/mg prot)	2.56 ± 0.22	4.59 ± 0.09 ^##^	3.17 ± 0.15 ^#^^*^
T-AOC (μmol/mg prot)	14.60 ± 1.75	2.40 ± 0.38 ^##^	6.55 ± 0.54 ^##^^*^
CAT (U/mg prot)	22.15 ± 1.74	7.93 ± 0.60 ^##^	20.24 ± 1.35 **
SOD (U/mg prot)	13.78 ± 0.42	8.21 ± 0.83 ^##^	11.25 ± 1.21 ^#^^*^
GSH-Px (U/mg prot)	20.40 ± 0.55	15.09 ± 1.29 ^##^	18.20 ± 0.51 *

# *P* < 0.05 and

## *P* < 0.01 vs. Control;

* *P* < 0.05 and

** *P* < 0.01 vs. Model.

### Western blot analysis

**Figure 7:**
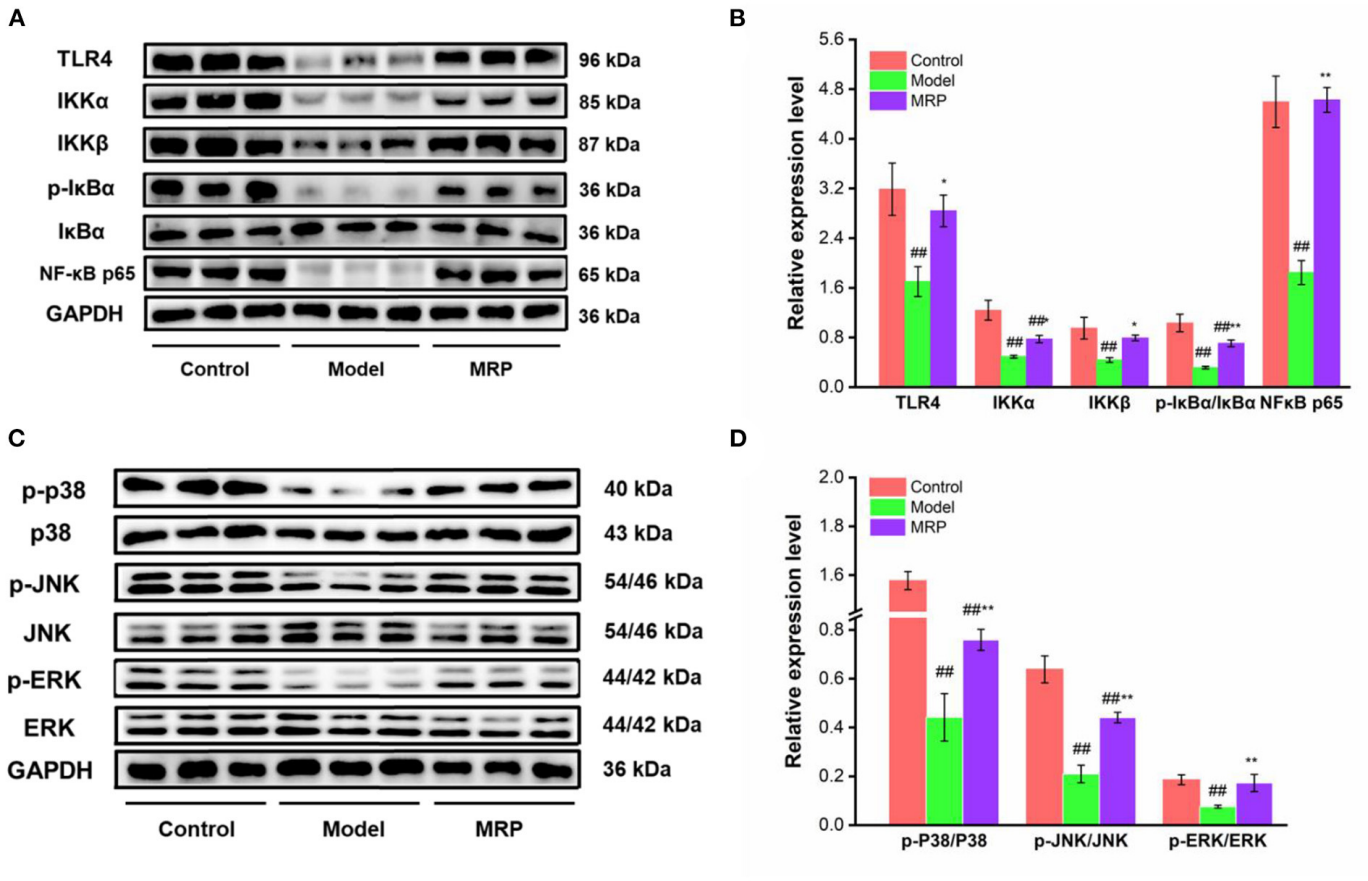
Effect of MRP on the protein levels in the NF-κB and MAPK pathways (*n* = 3). **(A)** Western blot of NF-κB pathway. **(B)** The protein levels of related proteins in the NF-κB pathway. **(C)** Western blot of MAPK pathway. **(D)** Protein levels of related proteins in the MAPK pathway. ^##^*P* < 0.01 vs. Control; *P < 0.05 and **P < 0.01 vs. Model.

The toll-like receptors (TLRs) family plays a key role in innate immunity (40, 41). When TLRs recognize relevant molecules, they activate downstream regulatory molecules such as NF-κB or interferon regulatory factor (IRF) to conduct immune signal transduction and induce and stimulate cells to produce effector molecules to kill foreign pathogens (13). TLR 4 is an important part of the TLRs family (42, 43). Numerous studies found that TLR 4 recognizes peptides and activates the downstream regulatory signal pathways, such as NF-κB and MAPK, to exert immunomodulatory effects (44, 45). Our study showed that the gray value of TLR 4 bands was obviously in control, while the gray value of TLR 4 bands in the model was decreased (Figure 7A). However, the use of MRP significantly increased the expression levels of TLR 4 (*P* < 0.05) as compared to the model (Figure 7B), indicating that MRP contained peptides that could bind to the TLR 4.

MAPKs and NF-κB are two classic pathways regulating the immune response (32, 46). Ma et al. indicated that collagen peptide-jackfruit juice could regulate immune response *via* the TLR 4/MAPKs/NF-κB pathways (32). He et al. showed that LMW peptides from *Mytilus coruscus* exhibited *in vitro* immunomodulatory effects *via* NF-κB/MAPK pathways (46). In the present study, these two classic pathways were used to investigate the immunomodulatory mechanism of MRP. Compared to the control, the IKKα (*P* < 0.01), IKKβ (*P* < 0.01), p-IκBα/IκBα (*P* < 0.01), NF-κB p65 (*P* < 0.01) expression levels were significantly downregulated in the model (Figures 7A,B). When treated with MRP, the IKKα (*P* < 0.05), IKKβ (*P* < 0.05), p-IκBα/IκBα (*P* < 0.01), and NF-κB p65 (*P* < 0.01) levels were significantly increased compared to the model (Figures 7A,B). Additionally, the p-JNK/JNK, p-ERK/ERK, and p-P38/P38 ratios were significantly decreased in the model compared to the control (Figures 7C,D, *P* < 0.01). When treated with MRP, these ratios were significantly upregulated compared to the model (Figures 7C,D, *P* < 0.01). Overall, MRP may regulate immune response *via* the TLR 4/MAPKs/NF-κB pathways, thus stimulating the production of cytokines to exert immunity effects.

### The potential mechanism of MRP for enhancing immunity

Based on the above results, the MRP has immunomodulatory activation in CTX-induced immunosuppressed mice. The immunomodulatory activation of peptides mainly depends on their structure-activity relationships, such as amino acid composition, sequence length, charge properties, hydrophilicity and hydrophobicity, and molecular structures (28, 46). The positively charged amino acids, such as Arg, Glu, or phosphoserine residues, may be recognized by receptors on the immune cell surface, promoting their immunomodulatory activation (28, 29). The contents of positively charged amino acids were enriched in MRP, which may be one of the main reasons for the immunomodulatory activation of MRP.

Previous studies have shown that active peptides can activate cellular receptor-mediated signaling pathways by binding to the immune cell receptors. For example, He et al. (44) showed that peptide (TQIDKVVHFDKLPGF) purified from enzymatic hydrolysates of duck egg ovalbumin could enhance phagocytosis capacity, and promote NO, IL-6, and TNF-α secretion in RAW 264.7 cells. Results of molecular docking indicated that TQIDKVVHFDKLPGF had a good affinity toward TLR 2 and 4. So, the short peptide contained in MRP may be bound directly to the expression receptor of immune cells, thereby activating the cell expression receptor-mediated related signaling pathway, which may be another reason for the immunomodulatory activation of MRP.

## Conclusion

**Figure 8:**
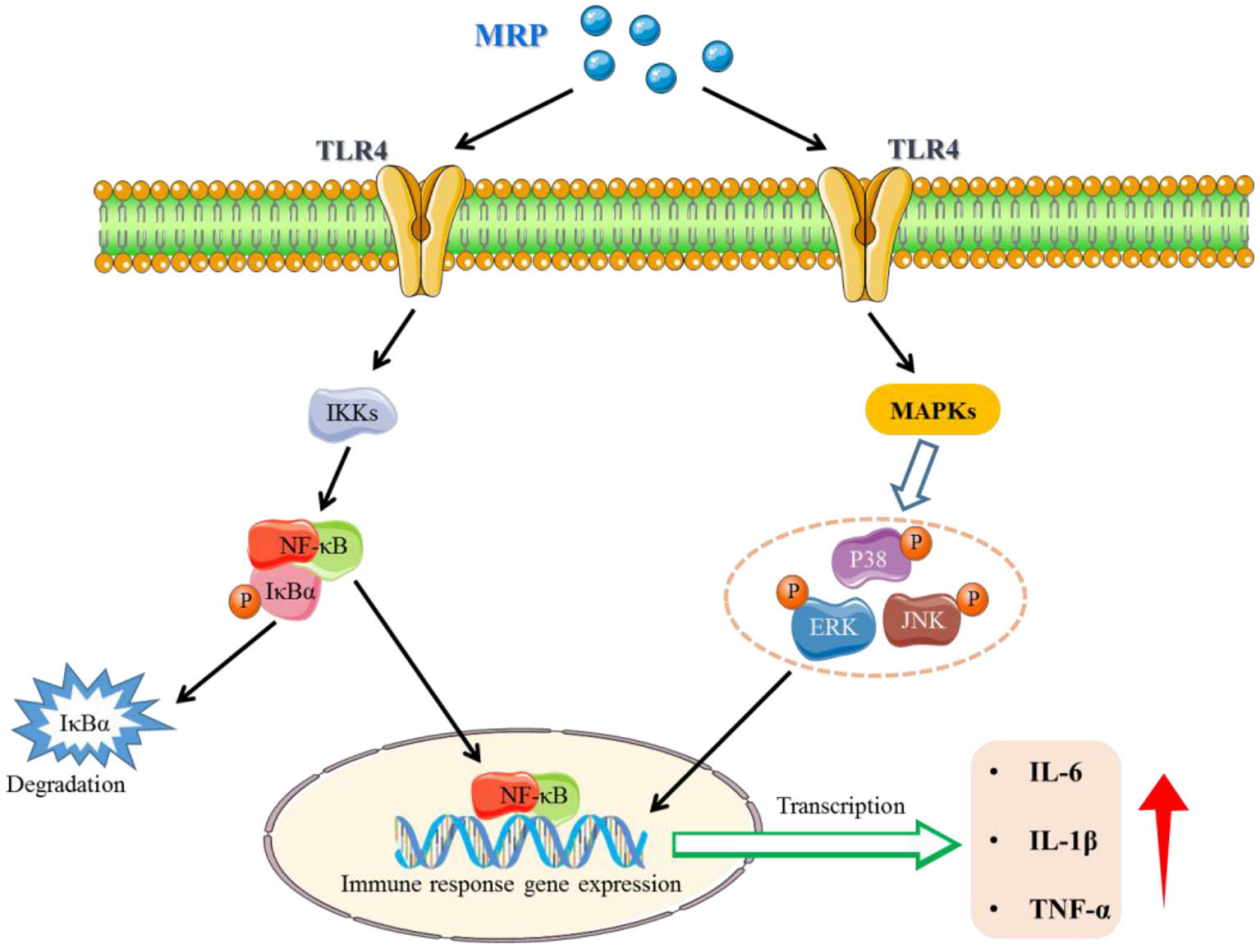
A potential signaling pathway involved in the immune-enhancement of CTX-induced immunosuppressed mice.

In conclusion, the LMW peptides (< 1 kDa, named MRP) from monkfish roe were prepared using enzymolysis and ultrafiltration. Our results indicate that MRP exhibits immunomodulatory effects by increasing the mice's body weight and immune organ index and improving morphological changes in the mice's spleen and thymus. Subsequently, this enhances the serum levels of cytokines (IL-6, IL-1β, and TNF-α) and immunoglobulins (IgA, IgM, and IgG) and activates the NF-κB and MAPK pathways (Figure 8). Our findings provide evidence for the immunomodulatory activation of MRP *in vivo* and will serve as a reference for the high-value-added utilization of fish roe.

## Data availability statement

The raw data supporting the conclusions of this article will be made available by the authors, without undue reservation.

## Ethics statement

The animal study was reviewed and approved by the Animal Ethics Committee of Zhejiang Ocean University (permission no. 2021016).

## Author contributions

JH and YT conceived the study, designed the project, revised the manuscript, and supervised the whole study. ZR, FY, SY, LB, and GJ experimented and analyzed the data. All authors contributed to the article and approved the submitted version.

## Funding

This work was financially supported by the Zhoushan Science and Technology Project (No. 2022C41004), the Basic Public Welfare Research Project of Zhejiang Province the Zhejiang Province (No. LGN20D060001), the Triple Agriculture Nine Aspects Cooperation Science and Technology Cooperation Program (No. 2022SNJF064), and the Zhejiang Xin-Miao Talents Program (No. 2021R411034).

## Conflict of interest

The authors declare that the research was conducted in the absence of any commercial or financial relationships that could be construed as a potential conflict of interest.

## Publisher's note

All claims expressed in this article are solely those of the authors and do not necessarily represent those of their affiliated organizations, or those of the publisher, the editors and the reviewers. Any product that may be evaluated in this article, or claim that may be made by its manufacturer, is not guaranteed or endorsed by the publisher.
